# Neurally adjusted ventilatory assist vs pressure support ventilation: short-term effects on shunt and dead space after cardiac surgery

**DOI:** 10.1038/s41598-025-33097-1

**Published:** 2025-12-21

**Authors:** Andreas Martinsson, Carl Lundholm, Sven-Erik Ricksten, Jonatan Oras, Jesper M. Magnusson, Andreas Wallinder, Johan Sellgren, Anders Thoren

**Affiliations:** 1 Department of Anaesthesiology and Intensive Care Medicine, Institute of Clinical Sciences, Sahlgrenska Academy, University of Gothenburg, Gothenburg, Sweden; 2 Department of Cardiothoracic Anaesthesiology and Intensive Care Medicine, Sahlgrenska University Hospital, Gothenburg, Sweden; 3 Department of Mathematics and Mathematical Statistics, Umeå University, Umeå, Sweden; 4 Department of Pulmonary Medicine, Sahlgrenska Academy, University of Gothenburg, Gothenburg, Sweden; 5 Department of Molecular and Clinical Medicine, Institute of Medicine, Sahlgrenska Academy, University of Gothenburg, Gothenburg, Sweden; 6 Transplant Institute, Sahlgrenska University Hospital, Gothenburg, Sweden; 7 Department of Anaesthesiology and Intensive Care Medicine, Sahlgrenska University Hospital, Gothenburg, Sweden; 8 Sahlgrenska University Hospital, Gothenburg, Sweden

**Keywords:** Cardiology, Diseases, Medical research, Physiology

## Abstract

Postoperative pulmonary complications, particularly atelectasis, are common after cardiac surgery and may contribute to impaired gas exchange or acute lung injury (ALI). Neurally Adjusted Ventilatory Assist (NAVA) delivers ventilatory support proportional to the patient’s respiratory drive, offering theoretical advantages over Pressure Support Ventilation (PSV), including improved synchrony, enhanced diaphragmatic efficiency, and reduced risk of ventilator-induced lung injury. However, comparative data on gas exchange, dead space, and regional ventilation during weaning after cardiac surgery remain limited. This prospective crossover study evaluated 12 mechanically ventilated patients with mild ALI following cardiac surgery across three ventilation phases: two PSV phases (PSV1 and PSV2) separated by a phase of NAVA. Intrapulmonary shunt fraction was calculated from measurements obtained via a Swan-Ganz catheter. Physiological dead space fraction (V_D_/V_T_) was assessed using three methods: the Bohr–Enghoff equation, end-tidal CO₂-derived alveolar dead space fraction (AVDSf-ET), and a novel time-to-volume converted capnographic approach (V_CAP-CALC_). Regional ventilation was assessed using electrical impedance tomography (EIT), and neuroventilatory efficiency (NVE) was calculated from diaphragmatic electrical activity (EAdi). Data were analyzed using linear mixed-effects models to account for repeated measures and within-subject variability. V_D_/V_T_ was significantly lower during NAVA compared with PSV1 and PSV2 when assessed by V_CAP-CALC_ (58.5% vs. 63.8% and 61.3%, respectively; *p* < 0.001). The PaO₂/FiO₂ ratio and NVE were significantly higher during NAVA (*p* = 0.01 and *p* = 0.037, respectively). No significant difference in pulmonary shunt fraction was observed. EIT revealed a modest increase in dorsal end-expiratory lung volume during NAVA, without redistribution of tidal volume or Center of Ventilation. The V_CAP-CALC_ method showed strong agreement with established dead space measures (R2 = 0.77–0.82) and demonstrated high repeatability (mean coefficient of variation 3.5%). NAVA is a safe and feasible ventilatory mode following cardiac surgery, associated with reduced dead space fraction, improved oxygenation and enhanced neuroventilatory efficiency. Given that shunt fraction remained unchanged, the observed improvement in ventilation–perfusion (V/Q) matching reflects a reduction in V_D_/V_T_. The potential implications for postoperative recovery and long-term outcomes merit evaluation in larger clinical studies.

ClinicalTrials.gov: NCT03217305. Initial Release 21/06/2017.

**Supplementary Information:**

The online version contains supplementary material available at 10.1038/s41598-025-33097-1.

## Introduction

Cardiac surgical patients are prone to develop postoperative atelectasis, which predispose to postoperative respiratory insufficiency and pneumonia^1,2^. Contributing factors include the inflammatory response to cardiopulmonary bypass, open chest surgery, ischemia–reperfusion injury, and the postoperative gravitational effects of the heart and mediastinum on adjacent lung segments^3,4^. Assisted ventilation facilitates weaning by reducing sedation needs and relying on the patient’s neuromuscular effort. Pressure Support Ventilation (PSV) delivers a preset level of support triggered by flow or airway pressure, while Neurally Adjusted Ventilatory Assist (NAVA) uses diaphragmatic electrical activity (EAdi), measured via an esophageal catheter, to provide proportional support based on neural respiratory drive. Compared to PSV, NAVA offers several advantages, including improved synchrony between drive and effort, reduced risk of over-assistance, lower sedation requirements, shorter weaning time, decreased need for post-extubation non-invasive ventilation^5^, and reduced mortality^6–9^. Although beneficial, NAVA is most likely still underutilized in clinical practice. Barriers include limited staff training, increased demands on physician bedside presence, catheter-related issues, challenges in selecting appropriate patients, and the fact that NAVA is a proprietary mode available only on ventilators from a single manufacturer requiring an additional module.

Comparative data on pulmonary shunt and dead space between PSV and NAVA during weaning after cardiac surgery are limited. We hypothesized that NAVA reduces both, as assessed by Swan-Ganz catheterization and time-to-volume converted capnography (V_CAP-CALC_).

## Materials and methods

### Study design

**Fig. 1:**
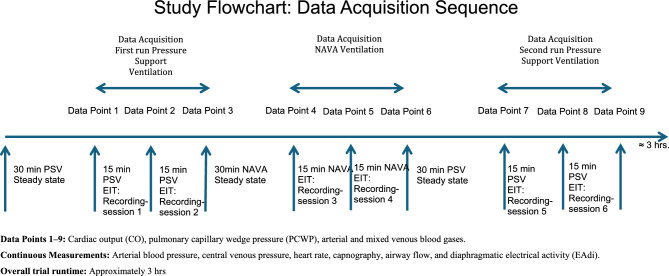
Study flowchart illustrating the data acquisition sequence across three ventilation phases (PSV1, NAVA, PSV2). Each phase comprised a 30-min steady-state equilibration followed by 30-min data acquisition, including three data points and two 2-min EIT recordings. The EAdi signal level was kept consistent throughout the trial, regardless of the ventilation mode. *PSV* Pressure Support Ventilation, *NAVA* Neurally Adjusted Ventilatory Assist, *EIT* Electrical Impedance Tomography.

This study was approved by the Regional Ethical Board in Gothenburg (Dnr:581–14) and conducted in accordance with the principles of the Helsinki Declaration. As the study inclusion criteria required prolonged postoperative mechanical ventilation under sedation, informed consent was obtained from the patients’ nearest relatives. The trial design was a prospective interventional within-subject crossover, comprising two PSV phases separated by an intervening NAVA phase. Each phase lasted 60 min, with 30 min for steady state followed by 30 min for data acquisition (Fig. 1). The ClinicalTrials.gov registration (NCT03217305) specified a 20-min acquisition period, which was extended to 30 min before study start. In accordance with EQUATOR guidelines for clinical trial reporting, key elements from the CONSORT 2010 Statement and the CONSORT Extension for Crossover Trials were applied (Supplement Fig. S1). Patients were enrolled between August 2018 and March 2022.

### Participant selection

Patient characteristics are summarized in Table 1. Included patients required invasive mechanical ventilation between postoperative days 3 and 10 following on-pump cardiac surgery. Eligibility criteria included stable ventilator settings in an assisted mode, a functioning NAVA catheter, and a consistent EAdi signal. Postoperative acute lung injury (ALI) was defined as a PaO₂/FiO₂ ratio ≤ 300 mmHg, corresponding to mild Acute Respiratory Distress Syndrome (ARDS) according to the Berlin definition^10^, and consistent with impaired pulmonary gas exchange not primarily attributable to left heart failure, pulmonary congestion, or fluid overload. Patients were excluded in cases of hemodynamic instability (norepinephrine infusion > 0.25 µg/kg/min, any inotropic support, or mixed venous oxygen saturation < 50%), deep sedation, contraindications to Swan-Ganz catheterization, or unreliable catheter readings.

**Table 1:** Continuous variables reported as mean ± standard deviation or median with interquartile range (IQR), depending on data distribution. Categorical variables reported as n (%).

Patient characteristics				
No of patients, n	12			
Age, years (IQR)	72.5 (65.3–76.5)			
Male/Female, n	9/3			
Hight (cm)	176.9 (6.4)			
Weight (kg)	72.8 (12.1)			
Body Mass Index (kg·m⁻^2^)	24.7 (2.6)			
Ideal body weight, male (kg)	75.4 (5.4)			
Ideal body weight, female (kg)	57.8 (5.7)			
Postoperative day (IQR)	5 (4–6)			
Type of surgery, n (%)	CABG + valve replacement, 6 (50)		Valve replacement, 3 (25)	CABG, 3 (25)
Main contributor to respiratory compromise, n (%)	Pneumonia, 5 (42)		Atelectasis w/wo pleaural effusion, 4 (33)	Aspiration pneumonitis, 3 (25)
Sedation, n (%)	Propfol and remifentanil, 8 (66)		Dexmedetomidine and remifentanil, 4 (33)	
Airway management, n (%)	Oral endotracheal intubation, 10 (83)		Percutaneus tracheostomy, 2 (17)	
Vasopressor treatment, n (%)	10 (83)			
Inotropic support, n (%)	0 (0)			
Hemodynamic parameters at start of trial				
Mean arterial pressure (mmHg)	73 (9)			
Heart rate (BPM)	74 (12)			
Central venous pressure (cmH_2_O)	12 (4)			
Mean pulmonary artery pressure (mmHg)	21 (5)			
Ventilatory settings and respiratory parameters	PSV1	NAVA	PSV2	
Pressure support (cmH_2_O)	8.1 (2.7)		8.0 (2.5)	
NAVA level (cmH₂O/µV)		1.0 (0.4)		
Positive end-expiratory pressre (cmH_2_O)	10.7 (1.8)	10.7 (1.8)	10.7 (1.8)	
Tidal volume (ml)	544 (138)	560 (148)	558 (129)	
Tidal volume / Ideal Body Weight, male (ml Kg^-1^)	7.5 (0.5)	7.6 (0.4)	7.6 (0.5)	
Tidal volume / Ideal Body Weight, female (ml Kg^-1^)	7.7 (0.7)	7.8 (0.8)	7.7 (0.7)	
Respiratory rate (RR)	18.5 (4.9)	18.0 (5.5)	17.9 (6.2)	
Minute ventilation (L)	9.5 (1.6)	9.6 (2.2)	9.4 (2.0)	
PaO_2_ (mmHg)	95.9 (17.8)	98.6 (15.2)	94.8 (17.7)	
Fraction inspired oxygen (FiO_2_)	0.38 (0.07)	0.38 (0.07)	0.38 (0.07)	
PaCO_2_ (mmHg)	37.0 (6.8)	37.1 (6.4)	37.3 (6.6)	
End tidal CO_2_ (mmHg)	31.1 (5.1)	31.7 (5.2)	31.3 (4.9)	

Body Mass Index was calculated as weight in kilograms divided by height in meters squared (kg·m⁻2). Ideal Body Weight (IBW) was calculated using the formula by Devine: IBW (male, kg) = 50 + 0.906 × (height in cm − 152.4); IBW (female, kg) = 45.5 + 0.906 × (height in cm − 152.4).

Vasopressor treatment refers to low-dose norepinephrine infusion (< 0.25 µg/kg/min). *CABG* Coronary Artery Bypass Graft, *NAVA* Neurally Adjusted Ventilatory Assist.

### Data acquisition

Hemodynamic and respiratory data were recorded using AcqKnowledge software (BIOPAC Systems, Inc., Goleta, CA, USA) connected via the MP150 interface and an intermediate analog-to-digital converter (Supplementary Fig. S2). Hemodynamic parameters were continuously sampled from the Philips IntelliVue Patient Monitoring System (Koninklijke Philips N.V., Amsterdam, The Netherlands). External events and manually obtained data–including arterial blood gas sampling, cardiac output and pulmonary capillary wedge pressure measurements, and initiation of EIT recordings–were annotated in AcqKnowledge as event markers, enabling precise temporal alignment with the continuously recorded signals (Fig. 1). Respiratory data, including airway flow, respiratory rate, and EAdi, were continuously collected from the Maquet Servo-u ventilator and integrated into AcqKnowledge via the Servo-Tracker interface and a digital-to-analog feed to the MP150 unit. Expired CO₂ was measured using a stand-alone side-stream capnometer (Datex Normocap, Datex Medical) connected directly to the MP150 unit. Because the capnometer operated independently and employed a side-stream sampling system, inherent delays and variable transit times precluded direct hardware temporal alignment. Instead, offline alignment was subsequently performed in AcqKnowledge by identifying the onset of the CO₂ decline at the transition from exhalation to inspiration and the sharp rise in inspiratory flow.

### Respiratory settings

Patients were positioned with the head elevated at 20–30° and sedated with either propofol–remifentanil or dexmedetomidine–remifentanil to maintain a Richmond Agitation–Sedation Scale (RASS) score of –3. This sedation level was selected to suppress coughing and spontaneous movements, which could compromise the accuracy of measurements. All patients received supportive invasive mechanical ventilation via either an endotracheal tube (size 7.0 for women and 8.0 for men) or a tracheotomy tube (size 8.0 for all). All patients received active heated humidification (MR850, Fisher & Paykel Healthcare, Auckland, New Zealand), heat and moisture exchanger (HME) filters were not used. The ventilator circuit was standardized for all patients and included the same Y-piece, corrugated circuit connector, and a CO₂ sampling adapter positioned proximal to the airway. PSV was delivered using the Servo-U ventilator (Maquet Critical Care, Solna, Sweden), with settings individually adjusted to achieve the most comfortable breathing pattern at the lowest effective pressure required to maintain normocapnia (PaCO₂ 35–45 mmHg), and a tidal volume (V_T_) of 6–8 mL·kg⁻1 ideal body weight. An inspiratory trigger sensitivity of -2 cmH₂O, along with titrated levels of PEEP and FiO₂ to maintain PaO₂ > 90 mmHg, were kept constant throughout the study protocol.

The NAVA catheter (EAdi Catheter, Maquet Critical Care, Solna, Sweden) was positioned in the esophagus via the nasogastric route and adjusted to optimize readings of the diaphragm’s electrical activity. The EAdi signal (µV) was titrated to target equal EAdi-max levels between PSV and NAVA at the start of each measurement period. This was accomplished by adjusting the NAVA level (cmH₂O/µV) and the pressure support level (cmH₂O), thereby ensuring equivalent respiratory drive across both ventilatory modes.

### EAdi metrics in NAVA ventilation

- Mean EAdi (EAdi_MEAN_, µV): Represents the mean electrical activity of the diaphragm across the entire respiratory cycle.
- Maximum EAdi (EAdi_MAX_, µV): The peak electrical activity of the diaphragm during inspiration; when multiplied by the NAVA level (cmH₂O/µV), it determines the peak inspiratory pressure delivered by the ventilator.
- EAdi Area Under the Curve (EAdi_AUC_, µVs/breath): Reflects the total electrical activity of the diaphragm throughout an entire respiratory cycle, providing an estimate of the overall diaphragmatic drive.
- Neuroventilatory Efficiency (NVE, mL/EAdi_MEAN_ µV): Represents the relationship between tidal volume and diaphragmatic electrical activity, providing an estimate of how efficiently neural respiratory drive translates into ventilatory output, reflecting the effectiveness of neuroventilatory coupling.
- Neural Triggering and Cycling: Inspiration begins when the EAdi signal exceeds 0.5 µV, and expiration is initiated when EAdi falls below 70% of its peak value; this threshold is referred to as the cycle-off criterion (Supplementary Fig. S3).

### Pulmonary artery catheterization

A Swan-Ganz pulmonary artery catheter (Edwards Lifesciences, Irvine, CA, USA) was inserted via the right internal jugular vein. Cardiac output (CO) was measured using bolus thermodilution. Each injection consisted of 10 mL of cold saline (~ 4 °C), drawn from an ice bath and injected smoothly over approximately four seconds, beginning at end-expiration. For each ventilation phase (PSV1, NAVA, PSV2), three data points were collected, including CO, pulmonary capillary wedge pressure (PCWP), and mixed venous oxygen saturation (mSvO₂) (Fig. 1). The average of the three values was used to represent each phase. Cardiac index (CI) was calculated as CO/Body Surface Area. The shunt fraction (Qs/Qt) was calculated using the Berggren equation^11^:

Qs/Qt = (CcO₂ – CaO₂) / (CcO₂ – CvO₂)

Where CcO₂ is the pulmonary capillary oxygen content, CaO₂ is the arterial oxygen content, and CvO₂ is the mixed venous oxygen content. Oxygen content was calculated as the sum of hemoglobin-bound oxygen and dissolved oxygen^12^. Pulmonary capillary oxygen content (CcO₂) was estimated using alveolar oxygen tension (PAO₂), which was derived from the alveolar gas equation^13^, assuming a respiratory quotient (RQ) of 0.8 and a dry barometric pressure of 713 mmHg. Full hemoglobin saturation was assumed for CcO₂ calculation. Venous oxygen content (CvO₂) was determined based on the mSvO₂ and venous partial pressure of oxygen (PvO₂), obtained from the pulmonary artery catheter.

### Calculating the pulmonary dead space fraction

The physiological dead space fraction (V_D_/V_T_) was derived using three different methods, with methodological details and derivations provided in the Appendix:

1. Bohr-Enghoff equation^14,15^ [PaCO_2_—PĒCO₂)/PaCO_2_]:

PĒCO₂ (mixed expired CO₂) was derived from V̇CO₂ (metabolic CO₂ production) using the calorimetric version of the alveolar ventilation equation (P̅ECO₂ = V̇CO₂ x 0.863 / VE)^16^. V̇CO_2_ was calculated from the RQ (assumed at 0.8) and V̇O_2_ (V̇CO₂=RQ x V̇O_2_). V̇O_2_ was calculated from the arterio-venous oxygen content difference^12^ (C_a-v_ O₂).

**Fig. 2:**
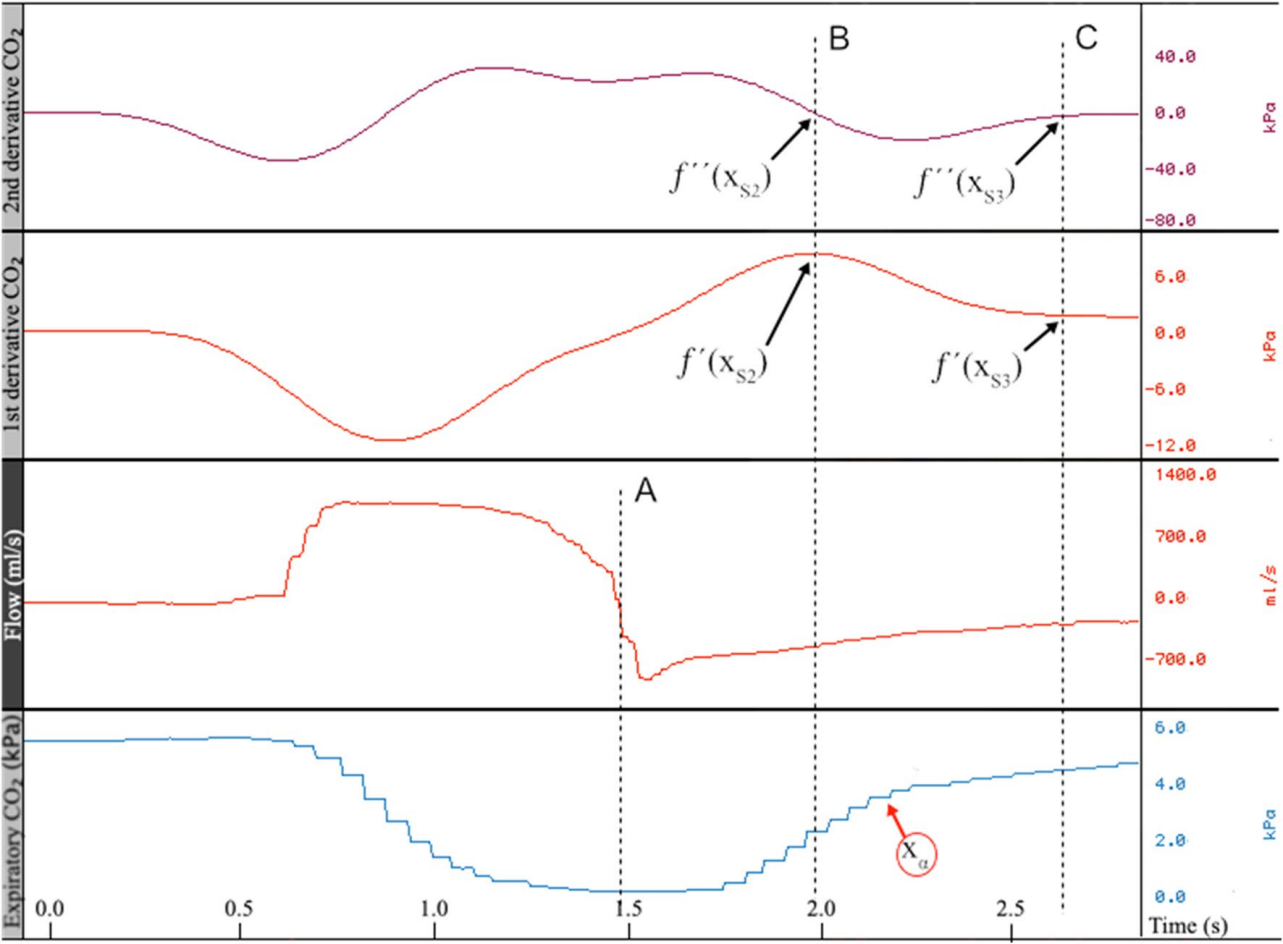
Expiratory CO₂ (kPa) time-aligned with airway gas flow over a single breath lasting 3 s. The upper two panels display the first and second derivatives of the CO₂ curve. Dashed line A marks the onset of expiration. Dashed line B indicates the transition from airway dead space exhalation to alveolar volume exhalation. Dashed line C indicates the point at which exhalation shifts from physiological dead space to gas originating from ventilated alveoli. The point labeled X_α_ represents the intersection of extrapolated straight lines approximating the mid-slope regions of Phase II (SII) and Phase III (SIII) of the capnogram.

2. End-tidal alveolar dead space fraction^17,18^ [AVDSf-ET, (PaCO_2_—PETCO_2_)/PaCO_2_]: The end-tidal CO₂ fraction (PETCO₂) was determined as the average of end-tidal values obtained from three 60-s recordings during each ventilation phase.
3. Time-to-Volume converted Capnography (V_CAP-CALC_):

A detailed description of this method is provided in the supplementary materials. In brief, time-integrals of respiratory flow was converted into volumes, time-aligned with the capnogram based on the onset of inspiratory flow. Onset of expiration is not visually detectable on the capnogram, hence defined as the point where flow transitioned from inspiration to expiration, as indicated by the dashed line A in Fig. 2. Accurate identification of this transition point is essential for subsequent analyses. Airway dead space was defined as the midpoint of Phase II of the capnogram, corresponding to Fowler’s original equal-area method^19^. The exact midpoint was determined as the inflection point along the Phase II curve, identified as the location of maximum inclination (i.e., the peak of the first derivative and the zero-crossing of the second derivative), and is shown by dashed line B in Fig. 2. The intersection of the extrapolated linear segments from the mid-portions of Phases II and III of the capnogram, referred to as slope II (SII) and slope III (SIII), defines the alpha angle, as outlined in several studies on the clinical interpretation of capnography waveforms^20,21^. The x-axis position of this intersection marks the transition from Phase II to Phase III and aligns with Tang et al.’s equal-area line, which defines physiological dead space using the same CO₂ reference as the Enghoff modification^22,23^. To objectively determine the x-axis position of the alpha angle (denoted x_α_, Fig. 2), we derived a combined equation based on the intersection of the linear equations describing SII and SIII. The value of x_α_ is given by:

$$x_{\alpha } = \frac{{f^{\prime}(x_{S2} ) \cdot x_{S2} - f^{\prime}(x_{S3} ) \cdot x_{S3} + f(x_{S3} ) - f(x_{S2} )}}{{f^{\prime}(x_{S2} ) - f^{\prime}(x_{S3} )}}$$

where:

- *f*´(x_S2_): The peak slope of CO₂, i.e., peak first derivative, was identified at the point where the second derivative, *f*''(xₛ₂), equals zero, marking the transition from positive to negative curvature and corresponding to the inflection point of Phase II.
- x_S2_: The x-axis position (time) at which *f*´(x_S2_) occurs.
- *f*(x_S2_): The PCO_₂_ value on the y-axis corresponding to x_S2_.
- *f*´(x_S3_): The slope of CO₂, i.e., peak first derivative, assessed at the mid-portion of Phase III, where the second derivative, *f*''(x_S3_), equals zero, indicating that the inclination is stable and approximately linear.
- x_S3_: The x-axis position (time) at which *f*´(x_S3_) occurs.
- *f*(x_S3_): The PCO_₂_ value on the y-axis corresponding to x_S3_.

### Calculating metabolic production of carbon dioxide

The Enghoff modification of the Bohr equation for calculating dead space requires the P̅ECO₂, traditionally obtained from expired gas collected in a Douglas bag^24^. Modern ventilators measure P̅ECO₂ via volumetric capnography. However, as neither method was available in the present study, three alternative approaches were employed to estimate V̇CO₂. Subsequently, P̅ECO₂ was calculated by applying the rearranged calorimetric version of the alveolar ventilation equation (P̅ECO₂ = V̇CO₂ × 0.863 / VE)^16^, with further methodological details provided in the Appendix.

1. Modified Harris-Benedict^25,26^ equation:

Estimates Resting Energy Expenditure (REE, kilocalories per day, kcal/day) based on an individual’s body height, weight, age, and gender. The Siddiqi modification^27^ further refines this estimate by accounting for prior surgery, trauma, infection, and fever. By applying Weir’s equation^28^ to the calculated REE and assuming a respiratory quotient of 0.8, V̇CO₂ can be calculated.

2. Arterio-Venous Oxygen Content Difference^12^ (C_a-v_ O₂):

V̇CO₂ is estimated by calculating the C_a-v_ O₂ and converting it to V̇CO₂ using an assumed RQ of 0.8 (V̇CO₂ = RQ x V̇O_2_).

3. Capnometry-Based (V_CAP-CALC_): Time-based capnometry was used to derive data analogous to volumetric capnography. Alveolar ventilation was estimated by subtracting physiological dead space from tidal volume, as inferred from capnography waveform analysis. V̇CO₂ was then calculated using the clinically adapted alveolar ventilation equation [V̇CO₂ = (PaCO₂ × V_A_) / 0.863].

### Electrical impedance tomography

#### Setup and data acquisition

A 16-electrode silicone EIT belt (Ref. 84 20 0-57/58/59, Dräger Medical, Lübeck, Germany) was positioned around the thoracic cage between the 6th and 7th intercostal spaces to avoid diaphragmatic interference. Placement was verified before each measurement. The belt was connected to a Pulmovista® 500 EIT device (Dräger Medical), sampling data at 40 Hz with a 50 beats·min⁻1 filter to attenuate cardiac motion artifacts. For each ventilation phase (PSV1, NAVA, PSV2), two 2-min recordings were acquired (Fig. 1). Data were analyzed using Dräger’s EIT Diag® software, with the PSV1 baseline recording serving as the reference for subsequent analyses.

#### Image reconstruction and analysis

The ventilated area was divided into equally sized ventral (non-dependent) and dorsal (dependent) ROIs, which were compared across ventilation phases. During baseline recording, tidal tissue impedance variation (∆Z) was calibrated against tidal volume, enabling subsequent calculation of dorsal tidal volume (V_T dorsal_) and dorsal end-expiratory lung volume change (∆EELV_dorsal_), expressed in arbitrary units (AU). The Centre of Ventilation (CoV) and Intra-Tidal Variation (ITV) display modes, along with validation metrics for EIT, have been previously described^1,29^.

#### Blood gas analysis

Arterial and venous blood samples were analyzed using a Siemens RAPIDPoint® 500 (Siemens Healthcare GmbH, Germany) to determine PaO₂/FiO₂ ratio, dead space fraction, and oxygen content.

### Primary and secondary outcomes

Primary outcomes include the comparison of pulmonary shunt fraction and physiological dead space fraction between PSV and NAVA. Secondary outcomes involved assessment of regional lung aeration by EIT (V_T dorsal_, ∆EELV_dorsal_, CoV, ITV), PaO₂/FiO₂ ratio, and NVE. Additional secondary analyses compared methods for estimating dead space fraction and V̇CO₂, including time-to-volume converted capnography (V_CAP-CALC_). Safety and feasibility outcomes were evaluated post-hoc to complement the predefined physiological endpoints.

## Data analysis

### Sample size calculation

The sample size was determined using a paired t-test to assess within-subject differences in shunt fraction between ventilation modes. The expected mean difference was estimated at 0.05, with a standard deviation of 0.05, based on pilot data. A two-sided significance level (α) of 0.05 and a statistical power of 80% (β = 0.20) were applied. Based on these assumptions, the required sample size was calculated to be 10 pairs.

### Statistical analysis

All statistical analyses were performed using SPSS (version 26, IBM Corp., Armonk, NY, USA). Patient characteristics and baseline values were summarized as mean ± standard deviation (SD) for normally distributed variables and as median with interquartile range (IQR) for non-normally distributed variables.

Comparisons between ventilation modes (PSV, NAVA, and a subsequent second run of PSV) were conducted using a linear mixed-effects model (LME). This was considered more appropriate than a standard repeated-measures ANOVA because it accounts for individual patient variability (with each patient serving as their own control), handles the non-independence of repeated measures (as each patient undergoes three measurements), and allows for modelling carryover effects (if NAVA influences the second PSV phase). When significance was detected, post hoc pairwise comparisons were performed using t-tests. A two-sided p-value < 0.05 was considered statistically significant. Assumptions of normality and homogeneity of variance for LME and paired t-tests were evaluated using the Shapiro–Wilk test and visual inspection of histograms, while Levene’s test was used to assess homogeneity of variance.

To compare different methods of calculating dead space, regression analysis was used to assess the predictive relationship between methods, while Bland–Altman analysis was performed to evaluate agreement.

### Coefficient of variation

The precision of the time-to-volume converted capnometry method were evaluated using the coefficient of variation (CV = [SD / Mean] × 100%). To assess technical precision under controlled conditions, we obtained 15 repeated measurements from a single patient, evaluating intra-individual repeatability. Three patients with 15 measurements each were included to capture inter-individual reproducibility. Mean CV was calculated as the average of individual CVs (∑CV / Number of Patients). An average CV below 5% was considered indicative of high precision across repeated trials and individuals.

## Results

### Patient demographics

Patient characteristics, including respiratory variables and baseline hemodynamics are summarized in Table 1. A CONSORT flow diagram is provided in the supplementary materials (Fig. S1).

### Primary outcomes

**Fig. 3:**
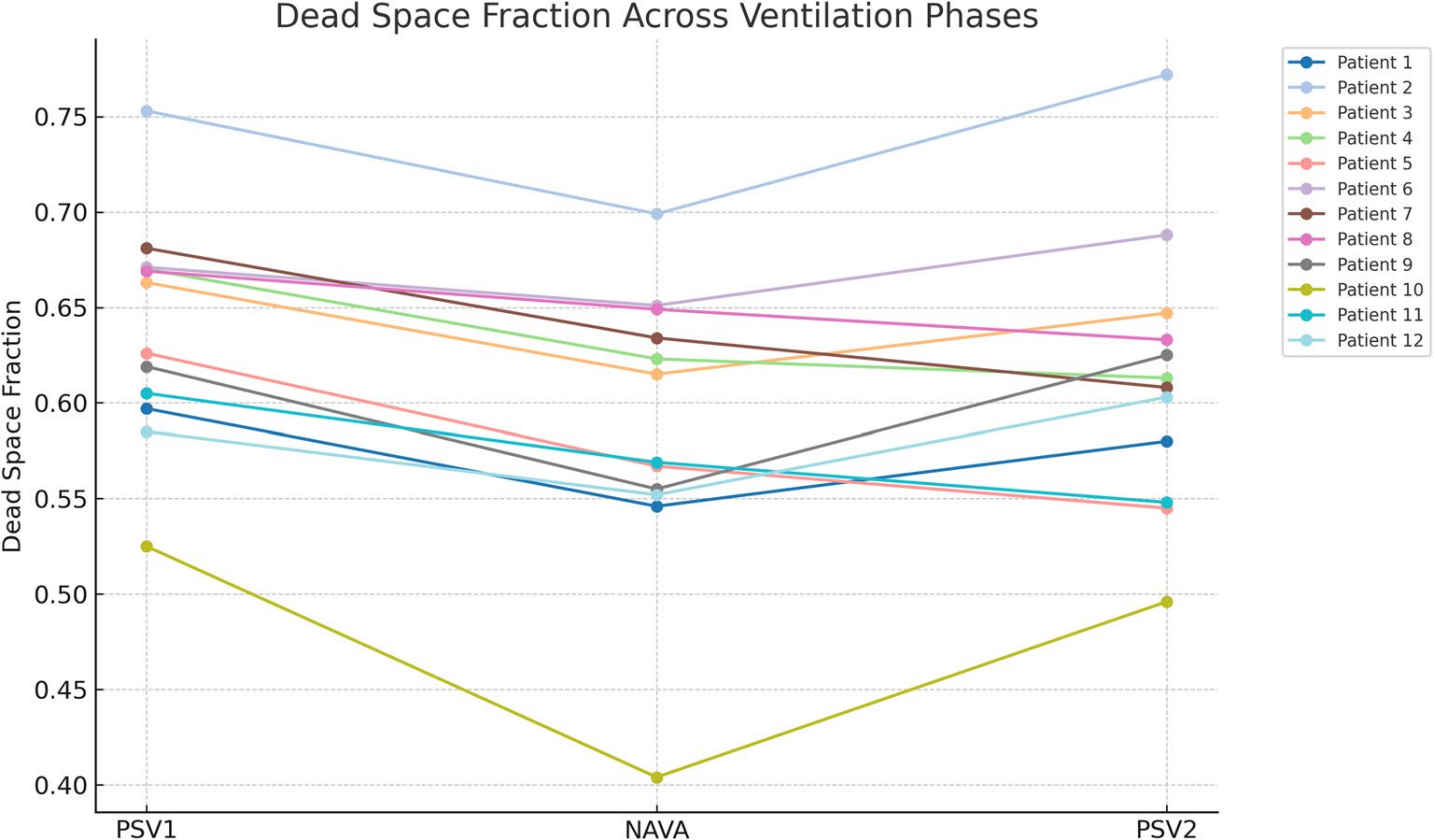
Individual capnometry-based dead space fraction values (V_CAP-CALC_) across three ventilation phases: First run Pressure Support Ventilation (PSV1), Neurally Adjusted Ventilatory Assist (NAVA), and second run Pressure Support Ventilation (PSV2). Each line represents one patient (n = 12), with values connected to illustrate within-subject changes across mode transitions.

Dead space fraction was significantly lower during NAVA compared to PSV, as assessed by V_CAP-CALC_ (58.5 vs. 63.8% in PSV1 and 61.3% in PSV2, respectively; *p* < 0.001, Table 2, Fig. 3). The mean paired difference between PSV1 and NAVA was − 5.0% (95% CI − 6.7 to − 3.3), and between NAVA and PSV2 + 2.5% (95% CI − 0.2 to + 5.1). No significant differences were observed in Bohr-Enghoff dead space or alveolar dead space fraction derived from end-tidal CO₂ (AVDSf-ET). Pulmonary shunt fraction showed no significant differences between ventilation modes (Table 3).

**Table 2:** Ventilatory parameters are presented as mean values ± standard deviations (SD) across the three ventilation phases (PSV1, NAVA, PSV2).

	PSV1	NAVA	PSV2	p-value	p-value	p-value	p-value
	Mean (± SD)	Mean (± SD)	Mean (± SD)	Linear mixed-effects	Post-hoc paired t-test: NAVA vs PS 1	Post-hoc paired t-test: PS 2 vs PS 1	Post-hoc paired t-test: PS 2 vs NAVA
PaO₂/FiO₂ ratio	266.3 (72.7)	279.0 (75.9)	263.3 (72.4)	.010	.030	.289	.009
EAdi mean (µV)	6.9 (1.8)	6.1 (1.4)	6.5 (1.8)	.089			
EAdi AUC (µVs/breath)	16.1 (8.9)	15.8 (9.1)	16.9 (11.4)	.580			
V_T_/EAdi (NVE, mL µV^-1^)	83.8 (28.6)	96.3 (31.3)	94.7 (36.8)	.037	.039	.043	.729
V_D_/V_T_ Bohr-Enghoff	0.535 (0.11)	0.535 (0.09)	0.528 (0.08)	.758			
V_D_/V_T_ AVDSf-ET	0.159 (0.04)	0.143 (0.06)	0.153 (0.05)	.522			
V_D_/V_T_ Capnography	0.638 (0.05)	0.588 (0.07)	0.613 (0.07)	< .001	< .001	.033	.070
V_D alveolar_/V_T_ Capnography	0.156 (0.018)	0.141 (0.025)	0.153 (0.018)	.007	0.023	0.397	0.018
V̇CO_2_ derived from RQ V̇O_2_	181.9 (31.6)	183.4 (26.7)	184.6 (27.3)	.770			
V̇CO_2_ derived from Modified Harris-Benedict	228.8 (32.0)	228.8 (32.0)	228.8 (32.0)	1.000			
V̇CO_2_ derived from capnography	170.0 (30.0)	175.3 (30.1)	171.0 (24.6)	.402			
EIT dorsal tidal volume (AU)	269 (67)	279 (81)	279 (88)	.767			
EIT ∆ EELV_dorsal_ (AU)		23 (39)	-43 (88)	.026	.079	.120	.036
EIT CoV (%)	49.9 (2.7)	49.7 (2.7)	49.8 (2.6)	.901			

P-values were calculated using a linear mixed-effects model (LME), with post hoc t-tests for significant effects. *AU* Arbitrary Unit, *AUC* Area Under Curve, *AVDSf-ET* Alveolar Dead Space fraction based on end-tidal CO_2_ fraction, *CoV* Center of Ventilation (above 50 = ventral dominance, below 50 = dorsal dominance), *EAdi* Electrical Activity diaphragm, *ΔEELV*_*dorsal*_ Dorsal End-Expiratory Lung Volume change, *NAVA* Neurally Adjusted Ventilatory Assist, *NVE* Neuro Ventilatory Efficiency, *PSV* Pressure Support Ventilation, *V*_*D*_*/V*_*T*_ Dead space fraction, *V*_*T*_ Tidal Volume.

**Table 3:** Circulatory parameters are presented as mean values ± standard deviations (SD) across the three ventilation phases (PSV1, NAVA, PSV2).

	PSV1	NAVA	PSV2	p-value
	Mean (± SD)	Mean (± SD)	Mean (± SD)	Mixed model ANOVA
Pulmonary shunt fraction, Qs/Qt (%)	14.5 (5.4)	14.2 (6.1)	15.2 (6.3)	.260
Cardiac index (L/min/m^2^)	2.28 (0.36)	2.33 (0.49)	2.33 (0.48)	.679
Cardiac output (L/min)	4.73 (0.87)	4.83 (1.11)	4.83 (1.08)	.659
Pulmonary capillary wedge pressure (mmHg)	13.5 (3.5)	13.4 (3.6)	14.3 (4.0)	.320
Pulmonary perfusion pressure (mmHg)	7.5 (2.4)	7.7 (2.7)	7.1 (2.6)	.540
Mixed venous oxygen saturation, Sv̅O₂ (%)	62.3 (6.7)	62.3 (6.6)	61.7 (6.3)	.637

P-values were calculated using a mixed-model ANOVA. *NAVA* Neurally Adjusted Ventilatory Assist, *PSV* Pressure Support Ventilation.

### Secondary outcomes

EELV_dorsal_ increased when switching from PSV to NAVA and decreased upon returning to PSV (increase of 23 AU and decrease of 43 AU, respectively; *p* = 0.026, Table 2). Ten of twelve patients showed an increase in ΔEELV_dorsal_ when switching from PSV1 to NAVA, and eight demonstrated a corresponding decrease when returning to PSV2 (Supplementary Fig. S4). No significant differences were observed in V_T dorsal_, CoV, or ITV (Supplementary Fig. S5). The PaO₂/FiO₂ ratio was significantly higher during NAVA compared to PSV (279.0 vs. 266.3 in PSV1 and 263.3 in PSV2, respectively; *p* = 0.01). The mean paired difference between PSV1 and NAVA was + 12.8 mmHg (95% CI + 1.5 to + 23.9), and between NAVA and PSV2 − 15.7 mmHg (95% CI − 26.7 to − 4.7).

NVE was also higher during NAVA (96.3 mL/μV vs. 83.8 mL/μV in PSV1 and 94.7 mL/μV in PSV2, respectively; *p* = 0.037, Table 2).

**Fig. 4:**
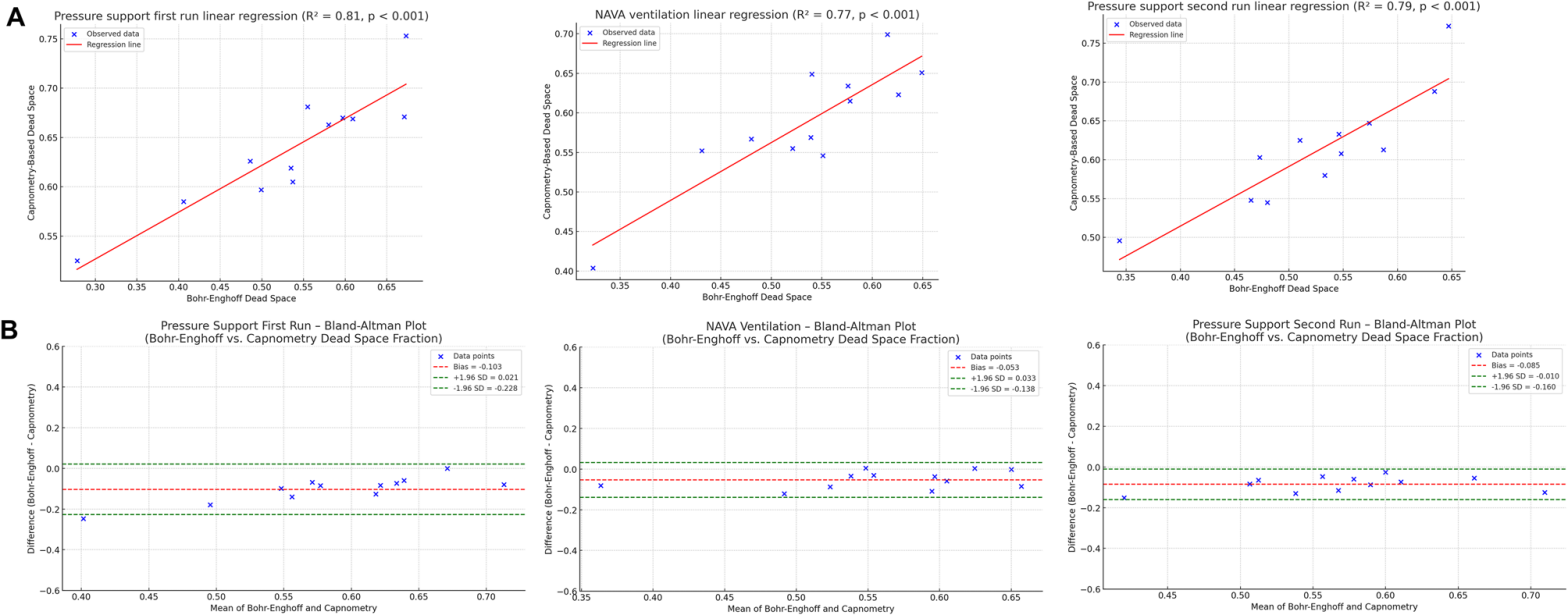
Panel **A**: Linear regression analysis plots illustrating the relationship between Bohr-Enghoff dead space (independent variable) and capnometry-based dead space (dependent variable) across ventilation phases. Blue points represent observed data, and the red line indicates the best-fit linear regression. The regression equations (slope and intercept) for each ventilation phase are as follows: PSV1: *y* = 0.48*x* + 0.38; NAVA: *y* = 0.58*x* + 0.29; PSV2: *y* = 0.60*x* + 0.30. These results indicate that for each unit increase in Bohr-Enghoff dead space, the corresponding increase in capnometry-based dead space ranges from 0.48 to 0.60 units, depending on the ventilation phase. Panel **B**: Bland–Altman plots comparing Bohr-Enghoff (α) vs capnometry-based (ß) dead space fraction (lower panel **B**), across ventilation phases. The X-axis represents the mean of methods α and ß for each breath, while the Y-axis shows the difference (α-ß), indicating how much method α differs from method ß. The red dashed line denotes the bias (mean difference), representing the average discrepancy between the two methods. The green dashed lines indicate the 95% limits of agreement (± 1.96 standard deviations), which encompass the range within which approximately 95% of the differences are expected to fall. No evidence of proportional bias or heteroscedasticity was observed in any of the plots.

Linear regression analysis revealed a strong correlation between the Bohr–Enghoff method, based on Swan-Ganz-derived measurements, and the V_CAP-CALC_ method (R2 = 0.77–0.82 across ventilation phases; p < 0.001, Table 4A, Fig. 4A). Bland–Altman plots demonstrated the average bias between the methods to be –0.098, –0.053, and –0.085 for PSV1, NAVA, and PSV2, respectively (Fig. 4B and supplementary Fig. S6). V_CAP-CALC_ yielded CVs of 2.44, 5.42, and 2.70% respectively, with an average CV of 3.52%.

**Table 4:** (A) Linear regression was used to assess the relationship between three different methods for calculating dead space. The Bohr-Enghoff method was used as the independent variable (X) and compared against the two other methods AVDSf-ET and Capnography. Comparisons were performed across all three ventilation phases (PSV1, NAVA, PSV2). (B) Linear regression was used to assess the relationship between three different methods for calculating metabolic CO2 production (V̇CO_2_). The RQ V̇O_2_ method was used as the independent variable (X) and compared against the modified Harris-Benedict equation and Capnometry-based method. Comparisons were performed across all three ventilation phases (PSV1, NAVA, PSV2). RQ V̇O_2_ method: V̇CO_2_ was calculated as RQ x V̇O_2_ where V̇O_2_ was derived from the arterio-venous oxygen content difference. *AVDSf-ET* Alveolar Dead Space fraction based on End-Tidal CO_2_, *NAVA* Neurally Adjusted Ventilatory Assist, *PS* Pressure Support, *RQ* Respiratory Quotient (assumed to be 0.8).

A				B			
	PSV1				PSV1		
Independent (X)	Dependent (Y)	R^2^	p-value	Independent (X)	Dependent (Y)	R^2^	p-value
Bohr-Enghoff	AVDSf-ET	0.01	0.86	RQ ∆Content O_2_	Modified Harris-Benedict	0.13	0.256
Bohr-Enghoff	Capnography	0.81	< 0.001	RQ ∆Content O_2_	Capnography	0.46	0.016
	NAVA				NAVA		
Independent (X)	Dependent (Y)	R^2^	p-value	Independent (X)	Dependent (Y)	R^2^	p-value
Bohr-Enghoff	AVDSf-ET	0.13	0.243	RQ ∆Content O_2_	Modified Harris-Benedict	0.18	0.171
Bohr-Enghoff	Capnography	0.77	< 0.001	RQ ∆Content O_2_	Capnography	0.50	0.010
	PSV2				PSV2		
Independent (X)	Dependent (Y)	R^2^	p-value	Independent (X)	Dependent (Y)	R^2^	p-value
Bohr-Enghoff	AVDSf-ET	0.11	0.283	RQ ∆Content O_2_	Modified Harris-Benedict	0.21	0.137
Bohr-Enghoff	Capnography	0.79	< 0.001	RQ ∆Content O_2_	Capnography	0.37	0.036

Linear regression analysis demonstrated a weak correlation between methods to calculate V̇CO₂ (Table 4B).

### Safety and feasibility

No adverse events related to the NAVA catheter were observed. Three patients were excluded due to insufficient EAdi signal quality.

## Discussion

The present study demonstrated significant physiological benefits of NAVA over PSV in post-cardiac surgery patients with mild ALI during the weaning phase. The short-term effects of shifting from PSV to NAVA included a reduction in physiologic dead space fraction, along with increased PaO₂/FiO₂ ratio and NVE, while pulmonary shunt fraction remained unchanged. To our knowledge, this is the first study in post-cardiac surgery ALI patients to evaluate the effects of NAVA on physiological dead space, intra-pulmonary shunt fraction, and neuroventilatory efficiency (NVE).

NAVA differs from conventional assist modes by utilizing diaphragmatic electrical activity (EAdi, in µV) as a surrogate for respiratory drive, rather than relying on flow or pressure triggers. This allows pressure delivery to be synchronized and proportionally adjusted to the EAdi signal during the inspiratory phase. The NAVA mode is previously described in detail^8,9,30–32^. Compared to PSV and other assist modes, NAVA offers several benefits during weaning in patients with ALI, ARDS, and chronic obstructive pulmonary disease, including improved patient–ventilator synchrony and comfort, reduced dorsal atelectasis through more effective activation of the dorsal diaphragm, and a lower risk of over-assistance and diaphragm atrophy^31^. Studies have also reported shorter duration of mechanical ventilation in selected ICU patients^9^, less need for non-invasive ventilation after extubation^5^ and even lower hospital mortality^9^.

### NAVA vs. PSV: pulmonary shunt

In this study, shifting from PSV to NAVA did not affect shunt fraction. Previous studies have linked NAVA with shorter durations of mechanical ventilation^9^, possibly through enhanced recruitment of dorsal atelectasis mediated by increased dorsal diaphragm activation. This mechanism is particularly relevant in supine ICU patients, as post-cardiac surgery atelectasis often involves the dorso-basal lung regions^1,33^. Although recruitment can improve V/Q matching and oxygenation, potentially reducing shunt, the lack of change in our study likely reflects an absence of severe lung injury, given the low baseline shunt fraction of approximately 14% and FiO₂ of 40%. The measurement technique may also have lacked sensitivity, or the improved PaO₂/FiO₂ ratio may rather been due to reduced dead space than shunt. A prior meta-analysis^9^ concluded that the most pronounced benefits of NAVA are seen in patients with more challenging weaning, unlike our population.

Early post-cardiac surgery, shunt fractions typically range from 14 to 20%^7,34^. In contrast, in a cohort of non-recruited patients with more severe ALI following cardiac surgery, shunt fractions approached 40% with FiO₂ levels around 70% and were partially reversible with prone positioning^35^. In this study, transitioning from PSV to NAVA did not change the shunt fraction but increased the PaO₂/FiO₂ ratio, likely reflecting improved V/Q matching. The modest yet reversible rise suggests enhanced gas exchange efficiency during NAVA, which, if sustained, may reduce oxygen or ventilatory requirements in more compromised patients. Improved oxygenation during NAVA has also been reported in a 24-h crossover study after abdominal surgery^36^.

### NAVA vs. PSV: physiological dead space

Physiological dead space, assessed via calculated volumetric capnography (V_CAP-CALC_), was 5% lower during NAVA compared with PSV in the present study. Although modest, this reduction most likely reflects partial recruitment of dorso-basal atelectasis, supported by an increase in dorsal end-expiratory lung volume (ΔEELV_dorsal_), indicative of improved dorsal aeration^1,33^. This recruitment enhanced V/Q matching and contributed to the improved oxygenation observed during NAVA. We speculate that the more homogeneous ventilation pattern associated with NAVA^37^, although not confirmed in our data (Table 2, Suppl. Fig. 4), reduces anterior alveolar overdistension and lower alveolar pressure on pulmonary capillaries, thereby enhancing capillary perfusion, reducing dead space, and improving the PaO₂/FiO₂ ratio. Such redistribution promoting more uniform lung stress and strain might also help mitigate ventilator-induced lung injury^38^–a hypothesis that warrants evaluation in future studies incorporating direct measures of these parameters.

The calculated mean differences and confidence intervals further substantiate the clinical relevance of this finding. The approximately 5% absolute reduction in dead space corresponds to an 8–9% relative improvement in ventilatory efficiency. Assuming that anatomical dead space remains constant, this reduction predominantly reflects a decrease in alveolar dead space, an effect of roughly 10%. Although numerically modest, changes of this magnitude are physiologically meaningful. In the landmark study by Nuckton et al., the difference in dead space fraction between survivors and non-survivors of early ARDS was 0.09 (0.54 vs 0.63)^39^. Thus, the present within-subject improvement during NAVA, supported by the reproducibility of the mean paired differences, reinforces the interpretation of a genuine enhancement in alveolar ventilation and V/Q matching.

Previous studies have reported physiological dead space values during mechanical ventilation shortly after cardiac surgery^40–42^, as well as after extubation^34^, but not during NAVA ventilation. In a study by Blankman and coworkers^40^, patients admitted to the cardiothoracic intensive care unit underwent lung recruitment, and at a PEEP level of 10 cmH₂O, the physiological dead space was 0.53 (V_CAP_), compared to 0.59 (V_CAP-CALC_) during NAVA in our study, supporting the interpretation of only mild ALI in the present cohort. Furthermore, V_D_/V_T_ values between 0.30 and 0.45 have been reported during controlled MV in the early postoperative period following cardiac surgery^34,42^.

Linear regression analysis comparing the calculated Volumetric Capnography (V_CAP-CALC_) and the Bohr-Enghoff Equation demonstrated a strong correlation. However, the Bohr-Enghoff estimates did not reach statistical significance, likely reflecting methodological limitations, as mixed expired CO₂ (P̅ECO₂) was indirectly derived from oxygen consumption and an assumed respiratory quotient of 0.8 (consistent with values reported after cardiac surgery^34^). As this approach assumes metabolic steady state and uniform gas exchange, minor deviations could have introduced variability and reduced sensitivity to within-subject changes. In contrast, volumetric capnography defines airway and alveolar dead space from the CO₂ waveform on a breath-by-breath basis, providing a more sensitive and physiologically responsive estimate. Notably, dead space fraction determined by volumetric capnography is regarded as the clinical gold standard^43,44^ and has been shown to predict mortality in early ARDS independently of oxygenation^45^. Accordingly, we consider V_CAP-CALC_ the most adequate and reliable method for this analysis.

### NAVA vs. PSV: electrical impedance tomography (EIT)

EIT is a well-established method for assessing regional lung aeration and is previously described in detail^1^. In the present study, EIT-derived variables including dorsal tidal volume, Center of Ventilation, and Intratidal Variation observed no redistribution toward the dependent lung regions during NAVA. This contrasts with previous findings of dorsal redistribution during NAVA in ALI patients treated in neurointensive care^46^ and general ICU settings^37,47^. While speculative, this discrepancy may reflect factors including deeper sedation^48^, milder ALI severity, shorter observation periods, or differences in patient selection. In contrast, ΔEELV_dorsal_, a reliable marker of dorso-basal aeration and atelectasis^1^, detected a modest increase during NAVA compared with PSV1, followed by a decrease during PSV2, indicating dorsal recruitment with subsequent derecruitment. Although absolute EELV_dorsal_ changes were small, their directionality was highly consistent: ten of twelve patients increased EELV_dorsal_ from PSV1 to NAVA, and eight decreased when returning to PSV2. The parallel improvement and decline in PaO_2_/FiO_2_ ratio reinforce that these shifts represented genuine physiological changes rather than measurement variability, suggesting that NAVA stabilizes and modestly recruits dependent lung regions. From a clinical perspective, the reproducibility and directionality of these within-subject responses are of greater interpretative value than their absolute magnitude, as they demonstrate a coherent and reversible physiological effect.

### NAVA vs. PSV: neuroventilatory efficiency (NVE)

In the present study, NVE was calculated as the ratio of tidal volume to the mean EAdi (V_T_/EAdi_MEAN_) per breath. This measure is considered to reflect diaphragm efficiency, meaning the coupling of drive (EAdi) to effort, resulting in synchronized diaphragm muscle force. NVE has previously shown best predictive power for extubation success in a general ICU population with diverse causes of respiratory failure^49^. In a mixed cohort of patients with ALI receiving prolonged mechanical ventilation, NAVA but not PSV improved NVE^50^. Additionally, monitoring V_T_/EAdi_MEAN_ during PEEP titration has been used to identify optimal PEEP levels associated with minimal diaphragmatic cost of breathing^51^. In the present study, NVE was 14% higher during the NAVA period compared with PSV1, supporting the interpretation of improved diaphragmatic efficiency. This enhanced coupling between respiratory drive and muscular effort likely reflect minor dorsal lung recruitment, resulting in an increase in local compliance. Notably, data on the effects of various modes of mechanical ventilation on NVE has not previously been presented following cardiac surgery.

### NAVA vs. PSV in tracheotomized patients

In a previous general ICU study, 99 patients with difficult weaning were randomized to receive either PSV or NAVA^6^. Interestingly, only the tracheotomized subgroup (n = 34) showed benefit from NAVA, including shorter weaning duration and more ventilator-free days at day 28. In our experience, tracheotomized patients require less sedation, potentially enhancing the effectiveness of NAVA. In the present study, only two tracheotomized patients were included, and sedation depth was more pronounced (median RASS –3) compared to RASS –2 reported by Ling and co-workers^6^. This difference may have influenced the limited physiological differences observed between PSV and NAVA. Unlike PSV, NAVA relies predominantly on diaphragmatic effort, which can be counteracted by too deep sedation^48,52^. Indeed, the advantage of NAVA has been attributed to reduced sedation requirements, as sedation can be more precisely titrated through continuous monitoring of respiratory drive^7^.

### Metabolic production of carbon dioxide

The comparison between estimates of V̇CO₂ revealed no significant correlations. This likely reflects the distinct physiological processes each method captures: arterio-venous oxygen content difference (C_a-v_ O_2_) quantifies total metabolic CO₂ elimination, whereas capnography measures the CO₂ actually exhaled at the airway opening. The adjusted Harris–Benedict estimate diverged from both measured methods, supporting their stronger basis in physiological measurement than theoretical estimation. However, comparisons must be interpreted with caution, as no absolute reference standard for V̇CO₂ was available.

### Limitations and strengths

The primary limitation of the study was the brief measurement periods, a common constraint in physiological studies that require time for equilibrium to be achieved. Nevertheless, switching from PSV to NAVA produced statistically significant changes, yielding new physiological and clinical insights. The within-person crossover design provided important advantages, including reduced inter-individual variability and the use of paired statistical analyses, which increased statistical power and allowed for a smaller sample size. However, this design also carries potential drawbacks, such as carry-over effects and the absence of blinding. In addition, the inclusion of patients with only mild ALI may have contributed to the modest differences observed between ventilation modes.

Finally, a limitation was the reliance on calculated rather than directly measured volumetric capnography, although the applied method demonstrated a low coefficient of variation. Specifically, there are several potential limitations related to the temporal alignment of the CO₂ signal from the standalone side-stream capnometer with the ventilator-derived flow signal. The flow signal was sampled at 200 Hz, whereas the CO₂ signal was acquired at 50 Hz, and side-stream sampling inherently smooths the capnogram^53^. In addition, the calculation of first and second derivatives involves an internal smoothing algorithm. The corrugated connector between the Y-piece and the CO₂ sampling line introduce a minor alignment bias, leading to slight overestimation of dead-space volumes. However, consistent use of the same alignment procedure across all subjects and ventilation phases minimized the potential bias in within-subject comparisons.

## Conclusions

NAVA is a safe and feasible ventilatory mode after cardiac surgery. Swan–Ganz catheterization enables calculation of pulmonary shunt and Enghoff’s physiological dead space, aiding weaning decisions. A novel method for converting time-based capnography to volumetric values may be more sensitive but is time-consuming. In this short-term physiological study, NAVA offered modest yet significant benefits over PSV, including reduced dead space fraction, improved lung oxygenation, and enhanced neuroventilatory efficiency. These findings suggest improved V/Q matching, primarily driven by dead space reduction. The potential implications for postoperative recovery and long-term outcomes merit evaluation in larger clinical studies.

## Supplementary Information

Supplementary Information.

## Data Availability

The datasets generated and analyzed during the current study are not publicly available due to institutional and ethical restrictions related to patient confidentiality. However, de-identified data are stored in a secure, controlled-access repository at Sahlgrenska University Hospital. Access to the data can be granted upon reasonable request from the corresponding author, subject to approval by the relevant institutional data protection authorities.
