# Supplementary material for: Neurally adjusted ventilatory assist vs pressure support ventilation: short-term effects on shunt and dead space after cardiac surgery
Source: Sci Rep. 2025 Dec 21;15:44234. doi: 10.1038/s41598-025-33097-1 (PMC12722728; doi:10.1038/s41598-025-33097-1)
Supplement: Supplementary file 1 — Supplementary Information. [file 41598_2025_33097_MOESM1_ESM.docx]

**Neurally Adjusted Ventilatory Assist vs Pressure Support Ventilation: Short-Term Effects on Shunt and Dead Space after Cardiac Surgery**

**Supplementary Material**

**Bohr Equation and the Enghoff Modification for the Measurement of Dead Space**

The physiologic dead space (V_D phys_/V_T_) is traditionally quantified using the Bohr equation^1^, which expresses the relationship between the difference in alveolar carbon dioxide fraction (FACO₂) and mixed expired carbon dioxide fraction (F̅ECO₂), normalized to the FACO₂. Conceptually, the Bohr equation assumes that a portion of the tidal volume (V_T_) does not participate in gas exchange (dead space, wasted ventilation), whereas the remainder is fully engaged in carbon dioxide exchange. This inherently simplifies the complex reality of pulmonary physiology by disregarding regional heterogeneity in ventilation-perfusion (V/Q) matching, where alveolar CO₂ concentrations may vary significantly across different lung regions. Consequently, the Bohr equation provides an idealized, yet practical, approximation of an intrinsically complex and spatially heterogeneous anatomic and functional environment.

The original formulation of the Bohr Equation^1^:

​​ V_T_ × F̅ECO₂ = (V_T_ −V_D_) × FACO_2_

Rearranged to express the dead space fraction:

V_D_ / V_T_ = (FACO_2_ - F̅ECO₂) / FACO_2_

Given the proportional relationship between gas fractions and partial pressures, the equation is conventionally expressed using partial pressures:

V_D_ / V_T_ = (PACO_2_ - P̅ECO_2_) / PACO_2_

Due to the difficulty of directly measuring alveolar CO₂, Enghoff^2^ modified the Bohr equation by replacing alveolar CO₂ pressure with arterial CO₂ pressure, which is readily obtained from blood gas analysis.

The Enghoff Modification^2^ of the Bohr-equation:

V_D_ / V_T_ = (PaCO_2_ - P̅ECO_2_) / PaCO_2_

The substitution of PACO₂ with PaCO₂ is theoretically justified by the assumption that arterial CO₂ reflects the average alveolar CO₂. However, this approach does not account for conditions that widen the PaCO₂–PACO₂ gap, such as low V/Q regions, intrapulmonary shunting, and diffusion impairment, leading to overestimation of V_D phys_/V_T_. As a result, Enghoff dead space is consistently larger than Bohr dead space.

**A Bedside Alternative for Alveolar Dead Space Estimation Using End-Tidal CO₂**

The original Bohr equation provides an idealized representation of dead space and depends on alveolar and mixed expired CO₂ measurements, which are difficult to obtain in clinical practice. A widely adopted simplification employs readily obtainable values, specifically the difference between arterial CO₂ and end-tidal CO₂ (FETCO₂), normalized to arterial CO₂, and is referred to as the End-Tidal Alveolar Dead Space Fraction (AVDSf-ET). This practical bedside method relies only on end-tidal CO₂ monitoring and arterial blood gas analysis, making it particularly useful for repeated assessments in critically ill, mechanically ventilated patients. AVDSf-ET has demonstrated strong correlation with conventional volumetric capnography^3^ and is associated with both the duration of mechanical ventilation and ICU mortality^4^.

Formulation of the AVDSf-ET Equation:

V_D_ / V_T_ = (PaCO_2_ - FETCO_2_) / PaCO_2_

**The Harris-Benedict Formula:**

Male REE (66,473 + (13,752 x weight in Kg) + (5,003 x height in cm) - (6,755 x age in years))

Female REE (655,096 + (9,563 x weight in Kg) + (1,85 x height in cm) - (4,676 x age in years))

The Siddiqi modification adjusts the calculated REE by applying specific multiplication factors based on increased metabolic demands:

Elevated temperature: ×1.13

Surgery: ×1.2

Trauma: ×1.35

Severe infection: ×1.6

**The weir equation:**

REE (kcal/day) = 1.440 (3.9 x VO_2_ + 1.1 x VCO_2_)

It can be rearranged using the respiratory quotient to express:

VCO_2_ = REE x RQ / (1.584 x (RQ + 3.545))

**Arterial-Venous Oxygen Content Difference:**

The concept of oxygen content (CO_2_) in blood was first described by Gustav Hüfner^5^ in 1894. The majority of oxygen in the blood is bound to hemoglobin, with a smaller fraction physically dissolved in plasma. Hüfner's constant quantifies the oxygen-binding capacity of hemoglobin, indicating how much oxygen each gram of hemoglobin can carry. The arterial-venous (A–v) oxygen content difference is determined by subtracting the venous oxygen content from the arterial oxygen content, providing a measure of tissue oxygen extraction.

Oxygen content was calculated as the sum of hemoglobin-bound oxygen and dissolved oxygen:

CO_2_ (mL O₂/dL blood) = Hb (g/L) × O₂ saturation (fraction) × 1.34 (Hüfner constant, mL O₂ / g Hb) + PaO₂ (mmHg) × 0.0031

**A Method for Converting Time-to-Volume Capnography and Standardizing Measurements**

The trial setup for our study involved side stream CO₂ sampling in conjunction with time-based capnography. By utilizing continuous in-circuit ventilator gas flow measurements, we converted time-integrals of flow into respiratory volumes. The flow curve was time-aligned with the capnogram based on the onset of inspiratory flow, identified by the sharp drop in CO₂, marking the transition from the alveolar plateau of phase III to baseline. The onset of expiration is not visually detectable on the capnogram, as the initial exhaled gas from the airway dead space contains no carbon dioxide. Hence, the onset of expiration was defined as the point where the flow transitioned from positive (inspiration) to negative (expiration), as indicated by the dashed line A in Figure 2. Accurately identifying this point is critical, as it marks the initiation for airway dead space volume tracing. The end of airway dead space expiration was defined as the midpoint of Phase II, corresponding closely to Fowler’s original equal-area method^6^. The exact midpoint was defined as the inflection point along the Phase II slope, identified as the location of the maximal inclination (corresponding to the peak of the first derivative and the zero-crossing of the second derivative), as indicated by dashed line B in Figure 2. Airway dead space was then defined as the volume of exhaled air from the start of expiration to this inflection point.

The intersection of the extrapolated linear segments from the mid-portions of Phases II and III of the capnogram, referred to as slope II (SII) and slope III (SIII), defines the alpha angle, as outlined in several studies on the clinical interpretation of capnography waveforms^7, 8^. The x-axis position of this intersection marks the transition from Phase II to phase III and aligns with the perpendicular line introduced by Tang et al. in their geometric equal-area analysis for determining physiological dead space^9, 10^. In this method, the equal-area perpendicular line bisects the capnogram such that the area under the curve to the left, representing expired CO₂ (VCO₂), is equal to the area to the right, which does not contribute to VCO₂. This implies that the entire area to the right of the perpendicular line represents expired volume containing alveolar CO₂ (according to the Bohr interpretation) or arterial CO₂ (per the Enghoff interpretation, as applied in Tang et al.'s original analysis). In contrast, the area to the left corresponds to the combined airway and alveolar dead space, i.e. physiological dead space, where no CO₂ is present. By defining physiological dead space in this way, alveolar dead space can be calculated by subtracting the known airway dead space (V_D alv_ = V_D phys_ – V_D aw_).

To objectively determine the x-axis position of the alpha angle (denoted x_α_, Figure 2), we derived a combined equation based on the intersection of the linear equations describing SII and SIII.

The straight-line equation is given by: y(x) = k_x_ ⋅ x + m_x_

SII(x): 𝑓´(x_S2_) ⋅ (x-x_S2_) + 𝑓(x_S2_)

SIII(x): 𝑓´(x_S3_) ⋅ (x-x_S3_) + 𝑓(x_S3_)

The value of x_α_ is given by the intersection point where the straight-line equations of Phase II and Phase III are equal, SII(x_α_) = SIII(x_α_). The equation is rearranged to solve for x_α_:

x_α_ = [𝑓´(x_S2_) ⋅ x_S2_ - 𝑓´(x_S3_) ⋅ x_S3_ + 𝑓(x_S3_) - 𝑓(x_S2_)] / [𝑓´(x_S2_) - 𝑓´(x_S3_)]


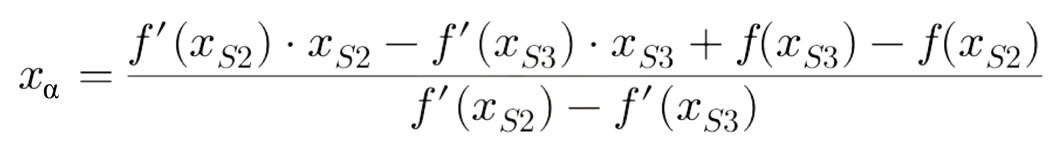


Where:

- 𝑓´(x_S2_): The peak slope of CO₂, i.e., peak first derivative, was identified at the point where the second derivative, 𝑓''(xₛ₂), equals zero, marking the transition from positive to negative curvature and corresponding to the inflection point of Phase II.
- x_S2_: The x-axis position (time) at which 𝑓´(x_S2_) occurs.
- 𝑓(x_S2_): The PCO_₂_ value on the y-axis corresponding to x_S2_.
- 𝑓´(x_S3_): The slope of CO₂, i.e., peak first derivative, assessed at the mid-portion of Phase III, where the second derivative, 𝑓''(x_S3_), equals zero, indicating that the inclination is stable and approximately linear.
- x_S3_: The x-axis position (time) at which 𝑓´(x_S3_) occurs.
- 𝑓(x_S3_): The PCO_₂_ value on the y-axis corresponding to x_S3_.

Derivatives were calculated in AcqKnowledge (Biopac Systems), and the zero-crossing of the second derivative was visually verified and manually adjusted to the nearest 0.02 s to ensure accurate alignment with the dashed reference lines B and C in Figure 2.

**References cited in Supplementary Material**

1 C B. Ueber die Lungenathmung *Skandinavisches Archiv für Physiologie* 1891; **1**: 236-68

2 H. E. Volumen inefficax. *Upsala Lakaref Forh* 1938: 191-218

3 Hardman JG, Aitkenhead AR. Estimating alveolar dead space from the arterial to end-tidal CO(2) gradient: a modeling analysis. *Anesth Analg* 2003; **97**: 1846-51

4 Bhalla AK, Chau A, Khemani RG, Newth CJL. The end-tidal alveolar dead space fraction for risk stratification during the first week of invasive mechanical ventilation: an observational cohort study. *Crit Care* 2023; **27**: 54

5 Hüfner G. Neue Versuche zur Bestimmung der Sauerstoffcapacität des Blutfarbstoffs. *Archiv für pathologische Anatomie und Physiologie und für klinische Medicin* 1894; **55**: 130-76

6 Fowler WS. Lung function studies; the respiratory dead space. *Am J Physiol* 1948; **154**: 405-16

7 Jaffe MB. Using the features of the time and volumetric capnogram for classification and prediction. *J Clin Monit Comput* 2017; **31**: 19-41

8 Herry CL, Townsend D, Green GC, Bravi A, Seely AJ. Segmentation and classification of capnograms: application in respiratory variability analysis. *Physiol Meas* 2014; **35**: 2343-58

9 Verscheure S, Massion PB, Verschuren F, Damas P, Magder S. Volumetric capnography: lessons from the past and current clinical applications. *Crit Care* 2016; **20**: 184

10 Tang Y, Turner MJ, Baker AB. A new equal area method to calculate and represent physiologic, anatomical, and alveolar dead spaces. *Anesthesiology* 2006; **104**: 696-700
